# An ultrastructural study of *Trichophyton rubrum* induced onychomycosis

**DOI:** 10.1186/s12879-015-1240-1

**Published:** 2015-11-17

**Authors:** Xueping Yue, Qing Li, Hongwei Wang, Yilin Sun, Aiping Wang, Qi Zhang, Cuiping Zhang

**Affiliations:** Department of Dermatology and Venereology, Beijing Tiantan Hospital, Capital Medical University, Beijing, 100050 P. R. China; Department of Ultrastructural Pathology, Beijing Neurosurgical Institute, Capital Medical University, Beijing, 100050 P. R. China; Department of Dermatology, Peking University First Hospital, Peking University, Beijing, 100034 P. R. China

**Keywords:** Onychomycosis, *Trichophyton rubrum*, Scanning electron microscopy, Transmission electron microscopy, Ultrastructure

## Abstract

**Background:**

*Trichophyton rubrum* (*T.rubrum*) caused onychomycosis is the most common nail fungal disease. The common diagnostic methods are direct microscopic examination and fungal culture. In this study we used scanning electron microscopy (SEM) and transmission electron microscopy (TEM) to study the subungual ultrastructural changes in *T. rubrum* induced onychomycosis.

**Methods:**

Six outpatients with onychomycosis were recruited and *T.rubrum* infection was confirmed by fungal culture. Six toenail samples were collected and prepared for SEM characterization. The cultured fugal colonies were prepared for SEM and TEM characterization.

**Results:**

1) SEM showed significant structural damages and the formation of a thin layer or a single layer of keratinocytes in all infected nail plates. Hyphae (piercing or penetrating keratinocytes layers), arthrospores and local bacterial aggregation were observed on the ventral surface of the nail plates. 2) SEM of the cultured fungal colony showed relatively straight, highly branched hyphae and microconidias; TEM showed branching hyphae that were composed of double-layer cell walls. Hyphae had nucleus, mitochondria, liposomes, lysosomes, scattered rough endoplasmic reticulum, myeloid bodies and aggregated ribosomes. There were high-density particles outside the hyphae.

**Conclusion:**

SEM showed a large number of hyphae penetrated the keratinocytes layer, suggesting that *T. rubrum* can cause severe damage to the stratum corneum. TEM showed the ultrastructural features of *T. rubrum*-induced infection before treatment.

## Background

Onychomycosis is a nail infection caused by dermatophytes and yeast. Dermatophytes are the most common pathogens. A nail infection caused by dermatophytes is called tinea unguis. *T. rubrum* is the most common pathogen among the dermatophytes. However, it is still unclear how *T. rubrum* invades the nail plate and what kind of ultrastructural changes occur after infection.

Aljabre et al. [1] reported that once in contact with stratum corneum, dermatophytes compete with the normal microbiota and cause adhesion. The dermatophyte arthrospores contact the stratum corneum and mediate the adherence process through the formation of fibrous flocs between the spore cell walls and keratinocyte membranes. Different dermatophytes exhibit different adherence abilities. For example, *Trichophyton mentagrophytes* (*T. mentagrophytes*) has a stronger adherence ability than *T. rubrum*. Meanwhile, Samdani et al. [2] showed that the fungal infection process is a combination of mechanical (hyphae invasion), chemical (microenvironment disruption) and biological (proteolytic enzyme) factors. Dermatophytes react with the substrates and produce a variety of proteases. These proteases hydrolyze keratin, collagen and elastin, which not only provide the necessary nutrients for the growth and metabolism of dermatophytes, but also facilitate the expansion and invasion of dermatophytes into the surrounding deeper tissues. Therefore, the proteases are considered the major dermatophyte virulence factor. Li et al. [3] measured the *in vitro* keratinase activity of the onychomycosis isolates and found that there was no significant difference in the keratinase activity between dermatophytes and non-dermatophytes, but the keratinase activity in *T. rubrum* was significantly higher than that in other tested fungi. However, keratinase activity in *T. rubrum* isolates was similar in clinical samples with different scoring clinical index for onychomycosis (SCIO). These results indicate that keratinases may be related to the incidence of onychomycosis, but keratinase alone cannot fully explain the pathogenesis of onychomycosis.

To date there are only a few reports on the subungual ultrastructural changes induced by onychomycosis. For example, Scherer et al. [4] reported the ultrastructural changes in two cases of onychomycosis caused by *T. rubrum* using scanning electron microscopy (SEM). Meyer et al. [5] studied the characteristics of onychomycosis caused by *Trichophyton mentagrophytes* using SEM. Harukuni UrabeHo et al. [6] reported the ultrastructural features of onychomycosis caused by *T.mentagrophytes* using transmission electron microscope (TEM). However, there is no report on the ultrastructural changes caused by *T.rubrum* using TEM. Therefore, in this study we used both SEM and TEM to investigate the ultrastructural changes in nail plate caused by *T.rubrum*, as well as to explore the underlying mechanisms of *T.rubrum* in the pathogenesis of onychomycosis.

## Methods

### Clinical data

Six outpatients (3 female and 3 male) with onychomycosis were recruited from October 2014 to January 2015 at the Capital Medical University, Beijing Tiantan Hospital dermatology clinic. There was no restriction on the age, gender and the duration of the disease. Inclusion criteria were as follows: nail plate thickening and color changing (pale yellow, yellow, white or gray-black), subungual debris accumulation, uneven surface or damaged nail plate; detection of fungus by direct microscopic examination and positive *T.rubrum* culture; no systemic and topical antifungal therapy in the past two years. Exclusion criteria were as follows: a history of diabetes, cancer, autoimmune diseases and other systemic diseases; infectious diseases within 1 year; hormones, immunosuppressants or antifungal drugs treatment within 2 year; oral antibiotics and other drugs treatment within 1 year. This study was approved by the Beijing Tiantan Hospital, Capital Medical University ethics committee. Study subjects were informed, consent and signed a written consent form.

### KOH staining

All surgical instruments were sterilized and the nail specimens were disinfected with 75 % ethanol. The debris from each nail was scraped and applied on a slide. A drop of 10 % potassium hydroxide (KOH) was added to each slide and the slides were observed under a Olympus optical microscope.

### Fungal culture

The nail specimens were disinfected with 75 % ethanol. The debris from each nail was inoculated on Sabouraud dextrose agar and incubated at 28 °C for 3–4 weeks. The conditions of the fugal culture were checked every 2–3 days and the types of fungi were identified after 3–4 weeks. A small portion of each colony was smeared on a slide, stained with lactic acid phenol Medan, and observed under a Olympus optical microscope.

### SEM nail sample preparation and observation

The samples were prepared in accordance with previous studies [4, 5, 7]. In brief, the samples were collected at the distal end of the infected nail. Each infected nail was cleaned with ethanol (Beijing Chemical Plant) and a specimen (width and length > 3 mm) was collected and dispensed in Eppendorf (EP) tube, fixed in 4 % paraformaldehyde (MERCH) and 2.5 % glutaraldehyde (Fluka) at 4 °C for 2 h. After washing, the specimen was post-fixed, dehydrated, displaced, dried and sprayed. The hexamethyldisilazane (Sinopharm Chemical Reagent Co. Ltd.) was used for chemical drying. The images were acquired with Hitachi TM-1000 (Hitachi, Japan) scanning electron microscope.

### SEM fungal culture colony specimen preparation

The samples were prepared in accordance with previous studies [4, 5, 7]. In brief, fungal colonies were cultured for 28 days and a rectangular specimen with agar (size of 1 × 0.8 cm) was collected from the edge of the cultured fungal colony using a sterile scalpel. The specimen was fixed in 4 % paraformaldehyde and 2.5 % glutaraldehyde at 4 °C for 2 h and processed as described above.

### TEM cultured fungal colony specimen preparation

The samples were prepared in accordance with previous studies [6, 8]. In brief, fungal colonies were cultured for 28 days and a square specimen with agar (size of 1 × 1 cm) was collected from the edge of the cultured fugal colony using a sterile scalpel. The specimen was immediately fixed in 2 % paraformaldehyde and 2.5 % glutaraldehyde, washed with 0.1 M cacodylic acid sodium salt erihydrate (pH7.4) (Beijing J & K Technology Co., Ltd.) and processed with Leica automatic tissue processor (Leica, EM, UC7). The specimen was post-fixed, washed, dehydrated, embedded with SPI812, positioned, sliced, stained, and observed under Hitachi H-7650 transmission electron microscope (Hitachi, Japan). The images were acquired with Gatan 832CCD camera (Gatan).

## Results

### The patients’ basic information

**Fig. 1:**
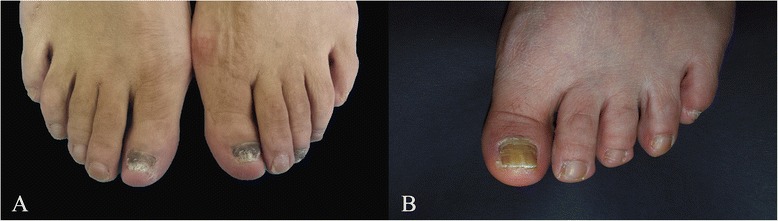
**a**. Case 1 had thickened nail plate, gray-black, visible subungual debris, and absence of distal nail plate. **b**. Case 2 had mild thickened nail plate, subungual yellow debris, yellow and uneven nail plate surface

Six outpatients with onychomycosis were recruited for this study. The patients were middle-aged men and women, average age of 55 years (46 to 61 years old), 2–8 toenail infections, with infection duration of 6 months to 15 years. The clinical manifestations of infected toenails were as follows: nail plate thickening, color changing (pale yellow, white, yellow or gray-black), subungual debris accumulation, uneven surface of nail plate (Fig. 1a, case 1), or nail plate breakage or absent (Fig. 1b, case 2). The patients’ basic information is shown in Table 1.

**Table 1:** The basic information of patients

Case number	Gender	Age	Diagnosis	Strains	Location	Lesions
1	Female	46	Onychomycosis	*T.rubrum*	Hallux	The whole nail was thickened and turned yellow, nail plate was absent, had subungual debris
2	Female	55	Onychomycosis	*T.rubrum*	Toenail	Part of the nail thickened and turned yellow, had subungual debris, no nail plate absence, uneven nail plate surface
3	Female	61	Onychomycosis	*T.rubrum*	Hallux	Distal portion of the nail turned white, had subungual debris
4	Male	52	Onychomycosis	*T.rubrum*	Toenail	The whole nail was thickened and turned yellow/black, had subungual debris
5	Male	57	Onychomycosis	*T.rubrum*	Hallux	Part of the nail turned yellow/black, part of the nail plate was absent
6	Female	60	Onychomycosis	*T.rubrum*	Toenail	The whole nail turned white, no nail plate absence, a little subungual debris

**Fig. 2:**
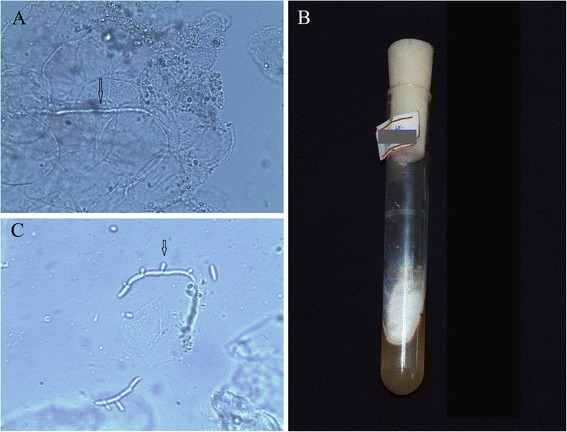
**a**. Light microscopic examination. Case 2 showed visible transparent hyphae in subungual debris (arrows) (light microscope × 400). **b**. Cultured fungal colonies in case 2 were identified as *T.rubrum*. **c**. *T. rubrum* cultured colonies from case 2. Microconidia  (arrow) were observed under the light microscope, (Medan stained, × 400)

Hyaline branching separate hyphae were detected in the six nail specimens under direct microscopic examination (Fig. 2a). Fungal cultures (Sabouraud dextrose agar, SDA) were positive (Fig. 2b). Microconidia were visible under an light  microscope (Fig. 2c). The fungal strain was identified as *T. rubrum*.

### SEM observation

**Fig. 3:**
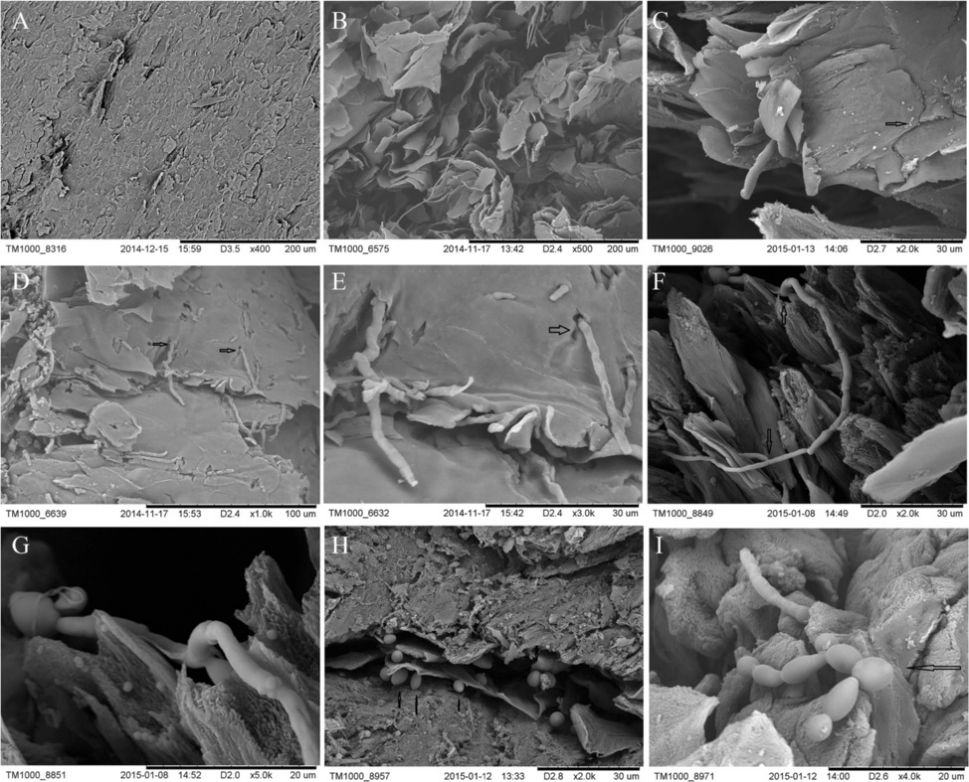
**a**. The ventral surface of the nail plate from a normal control. The nail plate was relatively intact without visible damage. (SEM, × 400). **b**. The ventral surface of the nail plate from case 1. The nail plate was significantly damaged with loose, layered and irregular shape. (SEM, × 500). **c**. The ventral surface of the nail plate from case 1. Hyphae were piercing through the thin layer of keratinocytes. Keratinocyte layer had scattered bacteria (arrow) (SEM, × 2000). **d**. The nail plate in case 1 showed significant damage, structural disorder and plenty of hyphae piercing through the thin layer of keratinocytes (arrow). (SEM, × 1000). **e**. The local amplification of Fig. 3d. There were visible hyphae piercing through the nail plate (arrows). Some of the hyphae were mellow, some were dry, had smooth surface without local destruction, no spores were visible. (SEM, × 3000). **f**. The image of case 2 showed budding and branching hyphae that piercing through the layered keratinocytes. The hyphae were complete and full. (SEM, × 2000). **g**. The local amplification of Fig. 3f. There were visible budding and branching hyphae. (SEM, × 5000). **h**. The image of case 3. There were visible subungual yeast-like cells. (SEM, × 2000). **i**. The image of case 4. There were subungual hyphae and pseudohyphaes-like features. (SEM, × 4000)

SEM indicated that the normal nail had an intact nail plate, tightly packed, and visible laminar at the ventral surface (Fig. 3a); whereas the infected nails had significantly damaged nail plates, dissociated layers, formation of a thin layer or single layer of keratinocytes. Meanwhile, hyphae (diameter 1 ~ 2 μm) were detected in all infected nails, often seen on the ventral surface of the nail plate. The images of case 1 showed a large amount of hyphae piercing and/or penetrated thin layers of keratinocytes. Most of the hyphae were straight and smooth. Some of the hyphae were dry and curved, but their surface was smooth and complete without local destruction. The images of case 2 showed budding and branching hyphae at the ventral side of the nail plate. (Fig. 3b-3g). The images of case 3 and 4 showed subungual pseudohyphae-like features and yeast-like cells (Fig. 3h-3i); while in images of case 5 and 6 showed local accumulations of bacteria. However, there was no* Candida* or bacteria observed under the light microscope or in the fungal culture.

### SEM observation of cultured fungal colonies

**Fig. 4:**
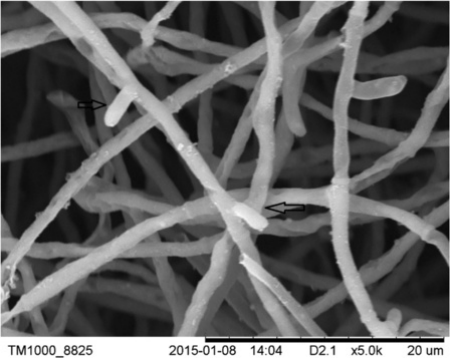
SEM observation of *T. rubrum* colonies. Most of the hyphae were straight and smooth, had intact surface without wrinkles. Some of the mycelia were partially dry. Scattered tiny particles were attached to the hyphae surface, had microconidia (arrows) and hyphae branches. (SEM, × 5000)

SDA cultured *T. rubrum* were mainly hyphae. Most of the hyphae were straight, smooth, branched, and intact surfaces without wrinkles. Some of hyphae were partially dry. Scattered tiny particles were attached to the hyphae surface. The hyphae had visible microconidias, but no macroconidia (Fig. 4).

### TEM observation of cultured fungal colonies

**Fig. 5:**
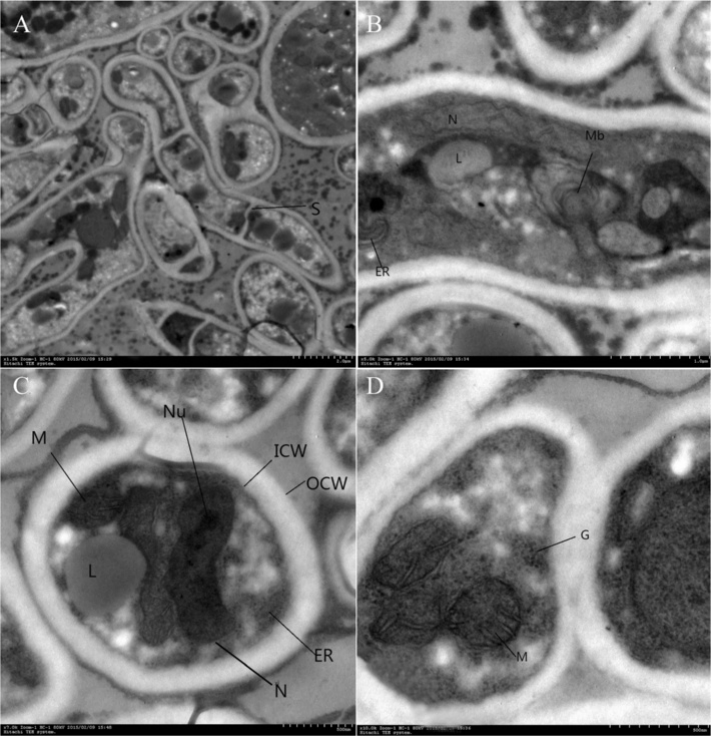
**a**. *T. rubrum* TEM observation. The image showed the longitudinal, coronal and chamfered surfaces of hyphae and longitudinal section of hyphae branches. The hyphae were constituted by bilateral cell walls, the outer cell wall (OCW) and the inner cell wall (ICW). The longitudinal section of hyphae branches showed visible septal (S), liposomes (L) and lysosomes (Ls). There were high-density particles outside the hyphae. (TEM, × 15000). **b**. TEM image of the longitudinal section of *T. rubrum* hyphae showed double-layer cell walls, visible nucleus (N), liposomes (L), the endoplasmic reticulum (ER) and myeloid body (Mb). (TEM, × 50000). **c**: TEM image of coronal section of *T. rubrum* hyphae showed double-layer cell walls, the outer cell wall (OCW) and the inner cell wall (ICW), nucleus (N), the nucleolus (Nu), liposomes (L), mitochondria (M) and the endoplasmic reticulum (ER). (TEM, × 70000). **d**: TEM image of coronal section of *T. rubrum* hyphae showed mitochondria (M) and ribosomes (G). (TEM, × 100000)

The longitudinal, coronal and chamfered surfaces and branches of hyphae were observed under TEM. The hyphae were constituted by bilateral cell walls, the outer cell wall (OCW) and inner cell wall (ICW). The longitudinal section of hyphae showed visible clear and complete septal (S). The hyphae had nucleus (N), nucleolus (Nu), mitochondria (M), liposomes (L), lysosomes (Ls), scattered rough endoplasmic reticulum (ER), myeloid body (Mb) and local accumulations of glycogen (G). The outside of hyphae had high-density particles (Fig. 5a-5d).

## Discussion

In this study we observed the subungual ultrastructural features of onychomycosis caused by *T. rubrum* under SEM and the ultrastructural features of *T. rubrum* under both SEM and TEM.

Our SEM findings are as follows: the nail plate was severely damaged, subungual structures were destroyed, *T. rubrum* were piercing through or penetrated thin layers of keratinocytes, budding and branching hyphae were visible. Our observations are consistent with two other cases of onychomycosis caused by *T. rubrum* in elderly patients reported by Scherer et al. [4]. However, we did not see  macrospores and arthrospores, which will be further investigated by increasing the sample size. In addition to Scherer et al. [4], Lee S et al. [9], Zhang et al. [10] and Jian et al. [11] all studied onychomycosis using SEM. They have observed hyphae and spores at the ventral side of the infected nail, but they have only provided very limited information on fungal morphology. In addition, they did not perform fungal culture; therefore, the strain of the fungi in their studies cannot be determined. Meyer et al. [5] observed SEM characteristics of onychomycosis caused by *T. mentagrophytes*, and their SEM results are similar to ours. For example, they also found that the hyphae penetrated the keratinocyte layers and hyphae were surrounded by a large number of fiber-like substances that might be soluble keratin. In our study we observed a large amount of hyphae penetrated keratinocytes layers which have not been reported before, but we did not see fiber-like substances, which needs to be further investigated. Moreover, we observed slender hyphae and isolated yeast-like cells, as well as pseudohyphae-like features in the subungual structures in the same patients, which have not been reported in any previous studies; therefore, our study is the first that reports this phenomenon. Oliveira et al. [12] reported the *Candida parapsilosis*-induced changes in hair and nails using a SEM. They found a large amount of subungual yeast cells but did not see pseudohyphal and keratinocyte layer penetration. Although pseudohyphal-like features and yeast-like cells were seen in our study, we didn’t see *Candida* in the fungal culture. Therefore, more studies need to be done to identify the presence of *Candida*. However, this phenomenon needs to be further investigated, since it will have impact on the choice of treatment drugs.

Previous studies reported scattered hyphae piercing or penetrated keratinocyte layers, however, in our study we found a large number of hyphae penetrated keratinocyte layers, suggesting *T. rubrum* exhibits strong adherence ability and secretes proteases that cause strong damages to the stratum corneum. These results may indicate that proteases break down keratin, cause keratinocyte layers to loosen and peel off, which form subungual debris and lead to nail plate thickening and degeneration. The destruction of nail plate facilities the diffusion and invasion of hyphae to deeper surrounding tissues and penetration of thin layer of keratinocytes. Muhsin et al. [13] determined the keratinase levels in 16 strains of fungi isolated from the experimental animals. Their results suggest that all the dermatophytes and most of the non-dermatophytes can produce keratinases. The keratinases level is high in dermatophytes, especially *T. mentagrophytes* var. Ireland and *Microsporum gypseum.* These reports suggest that we can compare the SEM ultrastructural changes with the keratinases levels in different types of fungal infections and explore the correlation between the keratinase levels and the pattern of hyphae diffusion, invasion and penetration in the keratinocyte layer. Due to the current limitations of the experimental conditions, we did not measure the keratinases levels in our patients, which will be performed in our future studies.

We observed typical budding and branching hyphae in our study, which is consistent with Rashid et al. [14]. It has been proposed that the adherence of dermatophytes spores and keratinocytes will cause germination. The longitudinal and lateral growth of the spores will form hyphae. The longitudinal growth of hyphae will penetrate deep stratum corneum, and laterally outward growth of hyphae will cause skin lesion expansion. In our study, we observed the adherence and germination of spores, as well as the hyphen penetration of the stratum corneum. Moreover, we also found that most of hyphae were straight and had intact smooth surfaces, with a diameter of 1 ~ 2 μm. Some of the hyphae were withered and bent, but the surface was very smooth and complete without local damage. These features have not been reported in previous literature. The differences in the hyphae morphology might be due to different nutritional status. Some hyphae have larger invasion space and rich nutrition, thus these hyphae are full and smooth; whereas, poor nutrition causes shriveled and bent in other hyphae. In this study, we also observed cultured *T. rubrum* colonies under SEM. We found that most of the hyphae were straight, smooth, branched, and had intact surfaces without wrinkles. Some of the hyphae had local shriveled areas. Scattered tiny particles were attached to the surface of the hyphae, which might be associated with fungal secretion. We only saw scattered microconidias, and did not see macroconidia. These results are consistent with previous studies [15, 16].

There are only a few TEM studies on the fungal morphology. Xu et al. [15] and our group [16] reported that *T. rubrum* hyphae have intact double-layer cell walls, uniformed cytoplasmic density, intracellular structures, such as mitochondria and rough endoplasmic reticulum. Urabe et al. [6] reported in details of *T. mentagrophytes* hyphae ultrastructure under TEM, which are similar to structures and organelles of *T. rubrum* hyphae observed in this study. Previous *in vitro* studies [8, 15, 16] investigated the structural changes before and after the treatment of *T. rubrum* infection. In our *in vivo* study we explored the detailed ultrastructural changes in nail plates and cultured fungal colonies from patients with *T. rubrum* infection using SEM and TEM before treatment, thus these results can be used in the future to compare the therapeutic effects before and after treatment in patients with *T. rubrum* infection *in vivo*.

This study has limitations. For example, we did not acquire simultaneous measurement of proteases levels in patients; therefore, we did not perform correlation analysis between the enzymes levels and the SEM results. On the other hand, we cannot identify if the pseudohyphal-like features and yeast-like cells were *Candida* or not. In our future research we will further investigate these issues.

## Conclusion

In summary, in this study we revealed the ultrastructural changes in the nail plate induced by *T. rubrum* infection and demonstrated that *T. rubrum* has strong adherent effects and causes severe damage to the nail plate. Meanwhile, our sturdy is the first to reveal that the ultrastructures of cell walls and intracellular organelles of *T. rubrum* hyphae under TEM.

## Abbreviations

EP: Eppendorf
ICW: inner cell wall
OCW: outer cell wall
SCIO: scoring clinical index for onychomycosis
SDA: sabouraud dextrose agar
SEM: scanning electron microscopy
TEM: transmission electron microscopy
